# Supplementary material for: From sequence to enzyme mechanism using multi-label machine learning
Source: BMC Bioinformatics. 2014 May 19;15:150. doi: 10.1186/1471-2105-15-150 (PMC4229970; doi:10.1186/1471-2105-15-150)
Supplement: Additional file 2 — Java code of ml2db. Additional file ml2db_code.tar.gz contains the Java source code to run the multi-label machine learning experiments and save the results to database. The code’s Javadoc is included. [file 1471-2105-15-150-S2.zip › additional file 2/ml2db/ml2db/doc/mulan/classifier/meta/HierarchyBuilder.html]

HierarchyBuilder


JavaScript is disabled on your browser.


- Overview
- Package
- Class
- Use
- Tree
- Deprecated
- Index
- Help

- Prev Class
- Next Class

- Frames
- No Frames

- All Classes

- Summary:
- Nested |
- Field |
- Constr |
- Method

- Detail:
- Field |
- Constr |
- Method


mulan.classifier.meta

## Class HierarchyBuilder

- java.lang.Object
- - mulan.classifier.meta.HierarchyBuilder

- All Implemented Interfaces:
  :   java.io.Serializable

  ---

    

  ```
  public class HierarchyBuilder
  extends java.lang.Object
  implements java.io.Serializable
  ```

  Class that builds a hierarchy on flat lables of given mulltilabel data.
  The hierarchy may be built with three methods.

  Version:
  :   0.1

  Author:
  :   George Saridis, Grigorios Tsoumakas

  See Also:
  :   Serialized Form

- - ### Nested Class Summary

    Nested Classes

    | Modifier and Type | Class and Description |
    | --- | --- |
    | `static class` | `HierarchyBuilder.Method` The different types of distributing labels to children nodes |
  - ### Constructor Summary

    Constructors

    Constructor and Description

    `HierarchyBuilder(int partitions,
    HierarchyBuilder.Method method)`

    Constructs a new istance based on given number of partitions and method
  - ### Method Summary

    Methods

    | Modifier and Type | Method and Description |
    | --- | --- |
    | `MultiLabelInstances` | `buildHierarchy(MultiLabelInstances mlData)` Builds a hierarhical multi-label dataset. |
    | `MultiLabelInstances` | `buildHierarchyAndSaveFiles(MultiLabelInstances mlData, java.lang.String arffName, java.lang.String xmlName)` Builds the hierarchy and constructs auxiliary files |
    | `LabelsMetaData` | `buildLabelHierarchy(MultiLabelInstances mlData)` Builds a hierarhy of labels on top of the labels of a flat multi-label dataset, by recursively partitioning the labels into a specified number of partitions. |
    | `static MultiLabelInstances` | `createHierarchicalDataset(MultiLabelInstances mlData, LabelsMetaData metaData)` Creates the hierarchical dataset according to the original multilabel instances object and the constructed label hierarchy |

    - ### Methods inherited from class java.lang.Object

      `equals, getClass, hashCode, notify, notifyAll, toString, wait, wait, wait`

- - ### Constructor Detail


    - #### HierarchyBuilder

      ```
      public HierarchyBuilder(int partitions,
                      HierarchyBuilder.Method method)
      ```

      Constructs a new istance based on given number of partitions and method

      Parameters:
      :   `partitions` - the number of partitions
      :   `method` - the partitioning method
  - ### Method Detail


    - #### buildHierarchy

      ```
      public MultiLabelInstances buildHierarchy(MultiLabelInstances mlData)
                                         throws java.lang.Exception
      ```

      Builds a hierarhical multi-label dataset. Firstly a random hierarchy is
      built on top of the labels of a flat multi-label dataset, by recursively
      randomly partitioning the labels into a specified number of clusters.
      Then the values for the new "meta-labels" are properly set, so that
      the hierarchy is respected.

      Parameters:
      :   `mlData` - the multiLabel data on which the new hierarchy will be built

      Returns:
      :   the new multiLabel data

      Throws:
      :   `java.lang.Exception`


    - #### buildLabelHierarchy

      ```
      public LabelsMetaData buildLabelHierarchy(MultiLabelInstances mlData)
                                         throws java.lang.Exception
      ```

      Builds a hierarhy of labels on top of the labels of a flat multi-label
      dataset, by recursively partitioning the labels into a specified number
      of partitions.

      Parameters:
      :   `mlData` - the multiLabel data on with the new hierarchy will be built

      Returns:
      :   a hierarchy of labels

      Throws:
      :   `java.lang.Exception`


    - #### buildHierarchyAndSaveFiles

      ```
      public MultiLabelInstances buildHierarchyAndSaveFiles(MultiLabelInstances mlData,
                                                   java.lang.String arffName,
                                                   java.lang.String xmlName)
                                                     throws java.lang.Exception
      ```

      Builds the hierarchy and constructs auxiliary files

      Parameters:
      :   `mlData` - the flat training data
      :   `arffName` - the name of the hierachical data
      :   `xmlName` - the filename for the hirearchy

      Returns:
      :   the hierarchical data

      Throws:
      :   `java.lang.Exception`


    - #### createHierarchicalDataset

      ```
      public static MultiLabelInstances createHierarchicalDataset(MultiLabelInstances mlData,
                                                  LabelsMetaData metaData)
                                                           throws InvalidDataFormatException
      ```

      Creates the hierarchical dataset according to the original multilabel
      instances object and the constructed label hierarchy

      Parameters:
      :   `mlData` - the original multilabel instances
      :   `metaData` - the metadata of the constructed label hierarchy

      Returns:
      :   the produced dataset

      Throws:
      :   `InvalidDataFormatException`


- Overview
- Package
- Class
- Use
- Tree
- Deprecated
- Index
- Help

- Prev Class
- Next Class

- Frames
- No Frames

- All Classes

- Summary:
- Nested |
- Field |
- Constr |
- Method

- Detail:
- Field |
- Constr |
- Method
